# The Influence of Soil Erodibility and Saturated Hydraulic Conductivity on Soil Nutrients in the Pingshuo Opencast Coalmine, China

**DOI:** 10.3390/ijerph19084762

**Published:** 2022-04-14

**Authors:** Mingjie Qian, Wenxiang Zhou, Shufei Wang, Yuting Li, Yingui Cao

**Affiliations:** 1 School of Land Science and Technology, China University of Geosciences (Beijing), Beijing 100083, China; zhouwenxiang@email.cugb.edu.cn (W.Z.); 3012200010@cugb.edu.cn (S.W.); 2012190028@cugb.edu.cn (Y.L.); caoyingui@cugb.edu.cn (Y.C.); 2 Key Lab of Land Consolidation, Ministry of Natural Resources of the People’s Republic of China, Beijing 100035, China

**Keywords:** soil erodibility, saturated hydraulic conductivity, soil nutrients, opencast coalmine, Loess Plateau

## Abstract

Soil erodibility (K factor) and saturated hydraulic conductivity (Ks) are essential indicators for the estimation of erosion intensity and can potentially influence soil nutrient losses, making them essential parameters for the evaluation of land reclamation quality. In this study, 132 soil samples from 22 soil profiles were collected to measure soil physicochemical properties (e.g., particle size distribution, bulk density and soil nutrient content) and calculate the K factor and Ks of reclaimed soils across the South Dump of the Pingshuo opencast coalmine in the Loess Plateau, China. Geostatistical analysis and the kriging interpolation were employed to quantify the spatial variations in the K factor and Ks in different layers. The results show that the K factor at 0–10 cm is obviously lower than that of other soil layers due to the external input of organic matter, while the Ks tends to decrease along with soil depth. Horizontally, the K factor at 0–10 cm and 50–60 cm shows a decreasing tendency from west to east, while that of other soil layers seems not to show any spatial distribution pattern along latitude or longitude. Meanwhile, the Ks at 0–10 cm presents a striped distribution pattern, while that of other soil layers shows a patchy pattern. On the other hand, the independent-sample *t*-test and Spearman’s correlation analysis were carried out to determine the effects of soil erodibility on total nitrogen (TN), soil organic matter (SOM), available phosphorus (AP) and potassium (AK). Overall, the K factor is negatively correlated with TN (r = −0.362, *p* < 0.01) and SOM contents (r = −0.380, *p* < 0.01), while AP and AK contents are mainly controlled by Ks. This study provides insight on the optimization of reclamation measures and the conservation of soil nutrients in reclaimed land of similar ecosystems.

## 1. Introduction

Opencast coal mining is a preferentially adopted exploitation method due to its cost-effective nature, despite the fact that it causes serious damage to original landforms through large-scale and high-intensity mining activities (e.g., stripping, excavation and transportation) [1]. Numerous studies report that the surface vegetation is thoroughly removed and the subsurface geological structures are largely reshaped during opencast mining, which triggers severe soil erosion in mining areas [2,3]. Moreover, Chinese opencast coalmines are primarily distributed in ecologically fragile regions (e.g., Inner Mongolia, Shanxi Province, Ningxia Province, Shaanxi Province and Gansu Province), where local ecosystems are increasingly threatened by soil and water losses [2]. The practice of land reclamation has been widely employed and developed all over the world as the most effective pathway for the ecological restoration of opencast coalmines; it is mainly characterized by soil reconstruction, landform remodeling and revegetation [4]. The optimal soil physicochemical and biological properties should be constructed for the optimal productivity of reconstructed soils. As such, it is vital for the implementation of land reclamation measures to understand and decipher the spatial distribution patterns of reconstructed soil properties [5]. For instance, reconstructed soils are always deficient in soil nutrients due to intensive mining activities and the removal of surface vegetation, making soil fertility an essential indicator for the quality of land reclamation. Moreover, large amounts of soil nutrients may be lost during the erosion process, highlighting the necessity of exploring the spatial distribution characteristics of soil erosion in the reclaimed land of opencast coalmines.

Characterized by the detachment and transportation of soil substances, soil erosion is regarded as a severe environmental problem that triggers serious soil degradation and threatens the service and function of ecosystems [6,7,8,9,10]. Soil erodibility (K factor) and saturated hydraulic conductivity (Ks) are essential parameters in influencing erosion intensity and have been widely employed to estimate soil loss rates during the erosion process [11,12,13,14,15]. The K factor reflects the inherent soil susceptibility to external erosivity forces and is reported to be mainly regulated by the structural stability of soil aggregates, while Ks can exert a non-negligible influence on the soil erosion process by regulating the infiltration and drainage of surface runoff [16,17,18]. Previous studies have reported that the K factor and Ks are largely influenced by surface vegetation, SOM and soil physicochemical properties (e.g., soil texture, bulk density and total porosity) [13,19,20,21,22,23,24]. Comprehensive and localized land management requires accurate spatial distribution information concerning soil properties, which highlights the importance of spatial analyses of soil erosion indicators. For instance, Wang et al. [20] quantified how vegetation restoration strategies influenced the distribution of the K factor in the Loess Plateau, and they concluded that the variations in K factor could be regulated by various factors, such as SOM content, plant litter density, soil bulk density (SBD) and biological crust thickness. Geostatistical methods can be effectively employed to estimate soil properties at unsampled locations based on a set of statistical tools [25]. Over the years, numerous interpolation techniques have been developed to quantitatively estimate the spatial distribution patterns of soil properties, such as pedo-transfer functions, inverse distance weighting (IDW), ordinary kriging (OK) and artificial neural networks [26,27]. The OK has been the most frequently adopted spatial interpolation method for soil properties because OK is considered the best linear unbiased predictor and can be easily conducted with high precision and accuracy [28,29,30]. Bonilla and Johnson [22] employed the OK interpolation to construct soil erodibility maps, and they found that soil erodibility was positively correlated with silt content but not correlated with SOM content in Central Chile. However, few studies have employed geostatistical methods to investigate the spatial variability of the K factor and Ks in reclaimed lands, despite the fact that their variability can reflect the overall quality of land reclamation efforts and guide the implementation of reclamation measures.

Soil nutrients are essential for the revegetation and ecological restoration of reclaimed lands; the dissolution and adsorption/desorption of these nutrients are strongly regulated by surface runoff, reclamation practices and the physicochemical properties of reclaimed soils [27,31,32]. Guan et al. [32] analyzed the spatiotemporal variations in soil nutrients under different land use types in reclaimed land of the Pingshuo opencast coalmine and concluded that the adopted reclamation measures significantly influence the evolution of reclaimed soil nutrients. Huo et al. [33] explored the interaction mechanisms among precipitation, surface runoff and soil nutrient losses, through which they found that high-intensity rainfall and runoff accelerate soil nutrient losses by increasing soil erosion. However, the effects of soil erodibility on soil nutrients have been rarely investigated, and several related studies concentrated only on the total nutrient content instead of the available nutrients that better reflect soil fertility [34,35]. Although Wang et al. [11] carried out laboratory simulation experiments to determine how soil erodibility affects the loss of available phosphorus and nitrogen, relevant research is still scarce, and research examining the effects of soil erodibility under natural conditions is required. Land reclamation measures can be greatly improved in order to reduce soil nutrient losses by optimizing the intrinsic soil properties if the interaction mechanisms between soil erodibility and soil nutrients can be better understood. Such an advance would provide scientific guidance for global land reclamation practices in similar ecosystems.

Overall, this study aims to: (1) analyze the spatial distribution characteristics of the K factor and Ks in different soil layers based on geostatistical analysis and kriging interpolation; (2) decipher the potential influencing factors for the spatial distribution patterns of the K factor and Ks; (3) explore how the K factor and Ks affect the accumulation and migration of total nitrogen (TN), SOM, available phosphorus (AP) and potassium (AK) in reclaimed soil profiles; (4) provide scientific guidance for land reclamation in opencast coalmine regions of similar ecosystems.

## 2. Materials and Methods

### 2.1. Study Area

The largest Chinese opencast coalmine is the Pingshuo opencast coalmine (112°11′–113°30′ E, 39°23′–39°37′ N). It is located in the east of the Loess Plateau and the north of Shanxi Province, where the soil erosion intensity is one of the highest in the world and the erosion rates vary from 5000 to 10,000 Mg km^−2^ yr^−1^ as a combined result of natural factors (e.g., intensive rainfall, erodible loess soil and a low vegetation coverage rate) and anthropogenic factors (e.g., mining, cultivation and overgrazing) [20,36]. Moreover, the ecosystem of the opencast coalmine was severely damaged due to intensive mining activities that hinder the restoration of vegetation and land reclamation in this region [2,37]. The present research was carried out in the South Dump of the Pingshuo opencast coalmine, which was employed as the open dump of the Antaibao opencast coalmine from 1985 to 1989 [38]. The study area is dominated by a semi-arid continental monsoon climate with a mean annual precipitation of around 450 mm, most (>65%) of which is concentrated from June to September [39]. Based on Chinese Soil Taxonomic Classification, the local soils are mainly composed of chestnut cinnamon soil and chestnut soil, which are of poor soil structure and low organic matter content and have limited ability to resist wind/water erosion [38]. The study area has suffered severe soil erosion due to the concentrated rainfall and loose soil structure; as a result, large amounts of soil nutrients essential to plants have been lost. According to previous studies and local long-term records, the soil erosion rate of the dump areas without reclamation (15,060 t·km^−2^·a^−1^) is much higher than that of the original Loess Plateau (10,120 t·km^−2^·a^−1^), while the erosion rate of the dump areas that have experienced 10-year revegetation is reduced to 3438 t·km^−2^·a^−1^ [2].

The South Dump is the earliest reclaimed region of the whole opencast coalmine and has experienced around 31 years of land reclamation, due to which the local ecological environment has been greatly improved [2]. The adopted land reclamation measures mainly comprise revegetation and soil reconstruction in the South Dump. The main plant configurations used for revegetation are *Hippophae rhamnoides*, *Pinus tabulaeformis*, *Ulmus pumila* and *Robinia pseudoacacia*. During the soil reconstruction process, various topsoil substitute materials (e.g., natural soils, coal gangue and rock fragments) were mixed to improve the soil productivity and landscape stability of the reclaimed land [38]. Different from natural soils, reconstructed soils are always characterized by high heterogeneity and bulk density.

### 2.2. Sampling and Analysis

**Figure 1:**
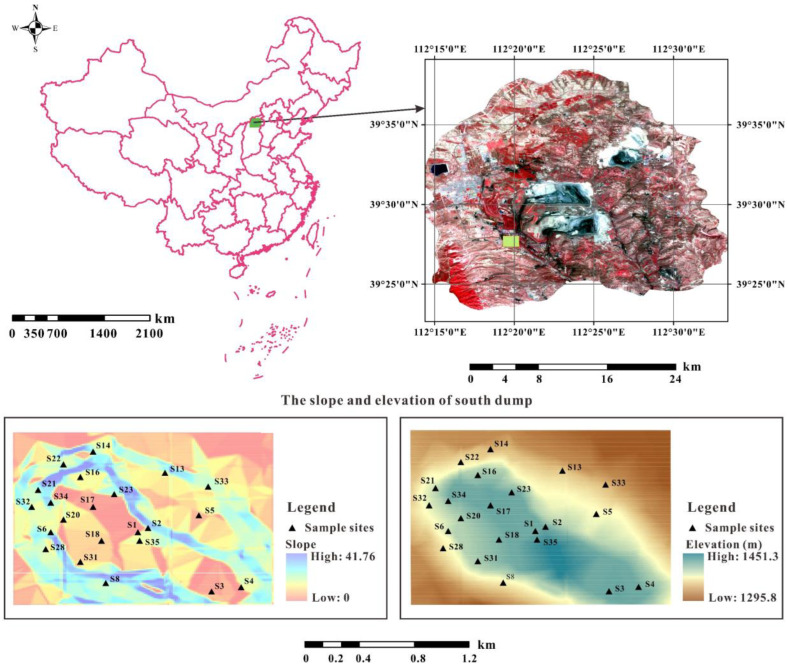
The location, slope, elevation and sample sites of the South Dump in the Pingshuo opencast coalmine, China.

A total of 132 soil samples were collected from 22 sample sites in May and August 2018 (Figure 1). These sample sites were selected because they represent the overall conditions in the South Dump (e.g., topography, vegetation types and soil conditions). Due to the exposure of hard rocks, only soil samples at 0–60 cm were collected and, based on our field observations, the sampling interval was determined as 10 cm at all sites. The quadrat was 10 m × 10 m on the platform in the first field sampling and 5 m × 5 m in the second field sampling. The size of the quadrat on slopes was adjusted to guarantee that the vertical projection area of the quadrat reached 10 m × 10 m or 5 m × 5 m [38].

After the collected soil samples were air-dried sufficiently, they were passed through a 2-mm sieve to remove unwanted materials (e.g., stones and plant residues) for subsequent laboratory analyses [40]. The contents of total nitrogen (TN) and soil organic matter (SOM) were measured based on the Kjeldahl and Walkley–Black methods [41,42]. Soil AK was extracted by NH_4_OAc solutions and determined by atomic absorption spectroscopic analyses, while AP was extracted by NaHCO_3_ solutions and measured using Olsen’s bicarbonate method [43,44]. After the pretreatment, particle size distribution (PSD) was determined by the laser particle size analyzer “Longbench Mastersizer 2000” (Malvern Instrument, Malvern, England) with a precision of 1%; the detailed experimental procedure we employed has been described by Liu and Han [45]. To obtain the herbaceous biomass, the plants on 1 m × 1 m quadrat at each sample site were randomly collected and weighted after they were sufficiently dried to a constant weight at 65 °C. These soil physicochemical properties were measured in the Beijing Academy of Agriculture and Forestry Sciences. Additionally, the data and experimental procedures for determining soil bulk density have been reported by Huang et al. [37].

### 2.3. Statistical Analysis

#### 2.3.1. Data Acquisition of the K Factor and Ks

The soil erodibility factor (K factor) has been widely employed to quantify the ability of soils to resist erosion processes and is mainly dominated by soil properties (e.g., organic matter, PSD and permeability) [46]. The K factor can be calculated by various models, such as the USLE model (Universal Soil Loss Equation) and the EPIC model (erosion productivity impact calculator) [47,48,49,50]. In this study, the K factor is calculated according to the EPIC model, the formula for which is listed as follows [51]:

(1) $$K=\left(0.3e^{\left[-0.0256Sa\left(1-\frac{Si}{100}\right)\right]}+0.2\right)\times {\left(\frac{Si}{Si+Cl}\right)}^{0.3}\times \left[1-\frac{0.25SOC}{SOC+e^{\left(3.72-2.95SOC\right)}}\right]\times \left[1-\frac{0.7Sa^{\prime }}{Sa^{\prime }+e^{\left(22.9Sa^{\prime }-5.51\right)}}\right]\;\;\;\;\;\;\;\;\;\;\;\;\;\;\;$$

where *Sa*, *Si* and *Cl* (%) represent the contents of sand, silt and clay, respectively. ${\mathrm{Sa}}^{\prime }$ (%) = 1 − *Sa*/100, while *SOC* (%) refers to the content of soil organic carbon that is derived from the measured SOM content (i.e., *SOC* = SOM × van Bemmelen factor) [52]. The van Bemmelen factor is a widely used conversion factor (0.58) to calculate *SOC* content based on measured SOM [53]. For the benefit of using an international unit (t ha h (ha MJ mm)^−1^), the calculated K factors are multiplied by a conversion factor (0.1317).

The saturated hydraulic conductivity (Ks) is an essential soil property for the prediction of soil water movement, which always triggers the redistribution of elements or nutrients in soil profiles [23]. The direct measurement of Ks is very time-consuming and expensive due to the strong nonlinearity of the unsaturated hydraulic conductivity function and water retention [54,55]. Moreover, the reconstructed soils in the South Dump are highly compacted and heterogeneous, which means that accurate measurement of Ks is very difficult for reclaimed land, and it is more appropriate to calculate Ks values based on simulation models. Various pedotransfer functions (PTFs) have been developed for the accurate prediction of soil hydraulic parameters, of which the Rosetta model based mainly on artificial neural network analyses (ANN) and the bootstrap resampling method are two of the most frequently employed models [56]. Rosetta1 was first proposed by Schaap et al. [57]; its reliability has been validated by various studies comparing measured and estimated Ks over the past two decades [11,58,59]. Moreover, the Rosetta model has been directly employed to calculate Ks and other soil parameters in the Loess Plateau [60], indicating that the model is qualified for the calculation of Ks in this study. The optimized Rosetta3 model was adopted to calculate the Ks (m/day) values based on the test data of PSD (percentages of clay, silt and sand) and soil bulk density by running the corresponding code in Python.

#### 2.3.2. Geostatistical Analysis

Geostatistical methods have been widely employed to describe the spatial variability of various soil physicochemical properties [61,62]. Previous studies have confirmed that the kriging interpolation method should be preferentially considered to predict the values of soil physicochemical properties at unsampled locations [23,48,63]. In this study, the ordinary kriging (OK) was adopted as the interpolation method to present the spatial distribution characteristics of the K factor and Ks, the optimal input parameters for which were obtained by the application of a semivariogram. The formula of the semivariogram is shown as follows:

(2) $$\gamma \left(h\right)=\frac{1}{2N\left(h\right)}\overset{N\left(h\right)}{\underset{i=1}{\sum }}{\left[F\left(x_{i}\right)-F\left(x_{i}+h\right)\right]}^{2}\;\;\;\;\;\;\;\;\;\;\;\;\;\;$$

where *γ*(*h*) refers to the semivariance for the given lag distance *h*, *N*(*h*) represents the total data pair number between sample sites separated by *h*, and *F*(*x_i_*) is the value of the variable *F* at *x*_i_.

#### 2.3.3. Data Validation

The cross-validation method was carried out to assess the quality of the OK interpolation in this study. This method picks out one datapoint from the sample pool each time and estimates this datapoint based on the model derived from the remaining data [48,63,64]. The errors between the predicted and actual values for all sample sites are calculated to assess the performance of the corresponding model (e.g., linear model, spherical model, exponential model and Gaussian model). The mean error (ME), absolute mean error (AME) and root mean square error (RMSE) are calculated as the evaluation indices for model stability. These indices can be calculated according to the following equations:

(3) $$\mathrm{ME}=\frac{1}{n}\overset{n}{\underset{i=1}{\sum }}\left(E_{i}-A_{i}\right)\;$$

(4) $$\mathrm{AME}=\frac{1}{n}\overset{n}{\underset{i=1}{\sum }}\left|E_{i}-A_{i}\right|\;\;$$

(5) $$\mathrm{RMSE}=\sqrt{\frac{1}{n}\sum_{i=1}^{n}{\left(E_{i}-A_{i}\right)}^{2}}\;\;\;\;\;$$

where *n* is the number of sample sites, $E_{i}$ refers to the estimated value and $A_{i}$ represents the actual observation value. Generally, ME, AME and RMSE values closer to zero denote better performance of the corresponding interpolation model. The geostatistical analysis and kriging interpolation in our study were carried out in GS^+^ (Gamma Design Software, version 9.0).

## 3. Results and Discussion

### 3.1. The Statistical Characteristics of the K Factor, Ks and Herbaceous Biomass

#### 3.1.1. Descriptive Statistics at Different Sample Sites

**Figure 2:**
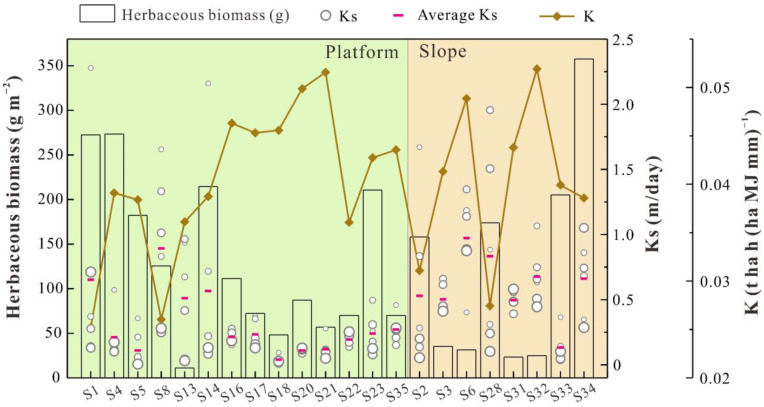
The distribution of K factor, Ks and herbaceous biomass at different sample sites. The K factor refers to the average value at 0–10 cm and 10–20 cm. The vertical distribution characteristics of Ks are presented by the bubble chart, where the larger bubbles represent deeper soil samples.

The average K factor at 0–10 cm and 10–20 cm, the surface herbaceous biomass and the vertical distribution characteristics of Ks at different sample sites are shown in Figure 2. The average K factor in topsoil (0–20 cm) ranges from 0.0252 to 0.0519 t ha h (ha MJ mm)^−1^ with an average value of 0.0403 t ha h (ha MJ mm)^−1^. The K factor is unevenly distributed among different sample sites, and the K factor values at sites S6, S20, S21 and S32 are much higher than those at S1, S2, S8 and S28 (Figure 2). As shown in Figure 2, the Ks values vary dramatically (0.006 m/day–2.278 m/day) and tend to decrease along with soil depth in most soil profiles. Overall, the Ks of topsoil is obviously higher than that of other soil layers at most sample sites, which results from the fact that deep soils are more compacted relative to topsoil. However, a vertical distribution tendency is not obvious at other sample sites (e.g., S16, S18, S20, S22 and S31), and the average Ks values are obviously different at different sample sites (Figure 2), which can be attributed to the high heterogeneity of reconstructed soils. Meanwhile, the herbaceous biomass varies dramatically (10.99–357.72 g m^−2^) at different sample sites and presents somewhat opposite distribution characteristics relative to the K factor in topsoil (Figure 2). For instance, a relatively high K factor always corresponds to an extremely low level of herbaceous biomass (e.g., sites S6, S21 and S32). On the one hand, higher erodibility triggers massive soil nutrient losses and reduces biomass in soils; on the other hand, SOM can act as an essential bonding agent that benefits the formation of soil aggregates and reduces soil erodibility [2,65,66]. Overall, herbaceous biomass is an important source of external organic matter input for local soils, and higher soil erodibility is adverse for the accumulation of SOM. A greater slope means higher runoff velocity and intensity, which accelerates the loss of fine soil particles and further decreases the K factor and increases the Ks in topsoil. To identify the potential influence of slope, the independent-sample *t*-test was employed to compare the difference between the K factor and Ks in the topsoil on platform and slope. The results show that there is no significant difference in the average K factor (*p* = 0.920) or Ks (*p* = 0.805) on the platform (K_mean_ = 0.0386 t ha h (ha MJ mm)^−1^, Ks_mean_ = 0.716 m/day; N = 14) and the slope (K_mean_ = 0.0382 t ha h (ha MJ mm)^−1^, Ks_mean_ = 0.642 m/day; N = 8), indicating that the influence of slope on the K factor and Ks can be ignored in the study area.

#### 3.1.2. Descriptive Statistics in Different Soil Layers

The descriptive statistics for the K factor and Ks in different soil layers are displayed in Table 1. The results of the Kolmogorov–Smirnov test indicate that both the K factor and Ks conform to a normal distribution (Table 1). The average K factor does not show a certain distribution pattern among different soil layers, although the average K factor at 0–10 cm is lower than that of other soil layers. Soil erodibility (K factor) is an essential indicator of the vulnerability of soil to erosion processes, and a greater K factor means a higher possibility of soil erosion [21,67,68]. The low K factor value of topsoil is most likely attributable to the fact that topsoil receives more external organic matter input, which can enhance water-stable aggregate contents [69,70]. Meanwhile, the average Ks value tends to decrease along with soil depth, which results from the fact that deep soil is more compacted and mainly characterized by low soil porosity and high bulk density. The coefficient of variation (CV) is frequently employed to describe the dispersion degree of variables, which categorizes variable types into weak variability (CV < 10%), moderate variability (10% < CV < 100%) and strong variability (CV > 100%) [32]. The evidence from CV indicates that the K factor presents moderate variability (16.42% < CV < 24.21%) while the Ks shows moderate (0–10 cm, 10–20 cm and 50–60 cm, 76.90 < CV < 94.75) to strong (20–30 cm, 30–40 cm and 40–50 cm, 104.18 < CV < 106.35) variability.

### 3.2. Geostatistical Analyses of K Factor and Ks

#### 3.2.1. Semivariogram Analyses

Semivariograms are frequently applied to investigate the spatial variability of soil physicochemical properties [64,71], the parameters of which are shown in Table 2 in this study. The Gaussian model is the best-fit model for the K factor in all soil layers except for 50–60 cm, for which the best-fit model is the linear model (Table 2). Similarly, the best-fit model for Ks is the Gaussian model at 0–10 cm, 30–40 cm and 40–50 cm, while it is the linear model at 10–20 cm and 50–60 cm and the exponential model at 20–30 cm. The determination coefficient (R^2^) ranges from 0.61 to 0.94, indicating that these optimal models are qualified for accurately describing the spatial variability of the K factor and Ks in different soil layers. The nugget (C_0_) and sill (C_0_ + C) of these models were obtained to describe the spatial structure of the K factor and Ks [64]. The ratio of C_0_/(C_0_ + C) is an essential indicator for the spatial dependence degree of soil properties, which categorizes the degree into strong spatial dependence (<25%), moderate spatial dependence (25–75%) and weak spatial dependence (>75%) [64,71]. According to the classification criterion, the K factors at 10–20 cm, 20–30 cm, 30–40 cm and 40–50 cm have strong spatial dependence, while the K factors at 0–10 cm and 50–60 cm are characterized by moderate to weak spatial dependence, indicating that soil erodibility in the middle soil layers is mainly regulated by intrinsic factors, while extrinsic factors exert a non-negligible influence on soil erodibility in the surface (0–10 cm) and bottom (50–60 cm) soil layers. Meanwhile, it can also be concluded that the levels of saturated hydraulic conductivity at 10–20 cm and 50–60 cm are mostly regulated by extrinsic factors, and the Ks levels in the remaining soil layers are mainly determined by intrinsic factors. The evaluation indices of model stability (ME, MAE and RMSE) are shown in Table 3. The ME, MAE and RMSE values for the K factor and Ks in different soil layers are all close to zero and remain stable (K factor: −2.90 × 10^−4^ < ME < 6.70 × 10^−4^, 5.86 × 10^−3^ < MAE < 9.45 × 10^−3^, 6.75 × 10^−3^ < RMSE < 1.11 × 10^−2^; Ks: −9.81 × 10^−3^ < ME < 1.24 × 10^−2^, 1.81 × 10^−1^ < MAE < 5.51 × 10^−1^, 2.34 × 10^−1^ < RMSE < 7.43 × 10^−1^), which indicates that the kriging interpolation results are credible for the K factor and Ks in different soil layers.

#### 3.2.2. Spatial Distribution Maps of K factor and Ks

**Figure 3:**
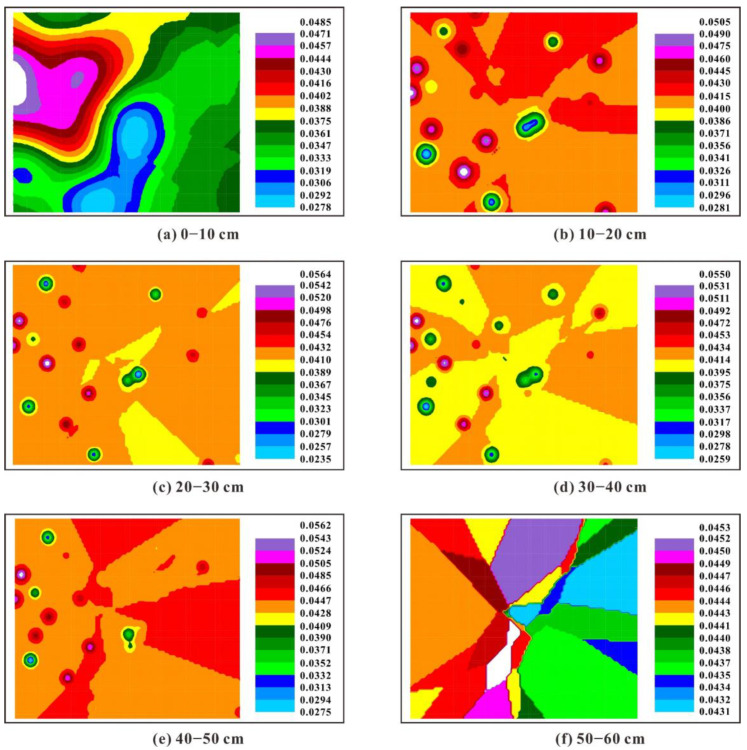
Spatial distribution maps of the K factor (t ha h (ha MJ mm)^−1^) in the South Dump based on the ordinary kriging interpolation. (**a**–**f**) represent the soil layers at different depths (0–10 cm, 10–20 cm, 20–30 cm, 30–40 cm, 40–50 cm and 50–60 cm).

**Figure 4:**
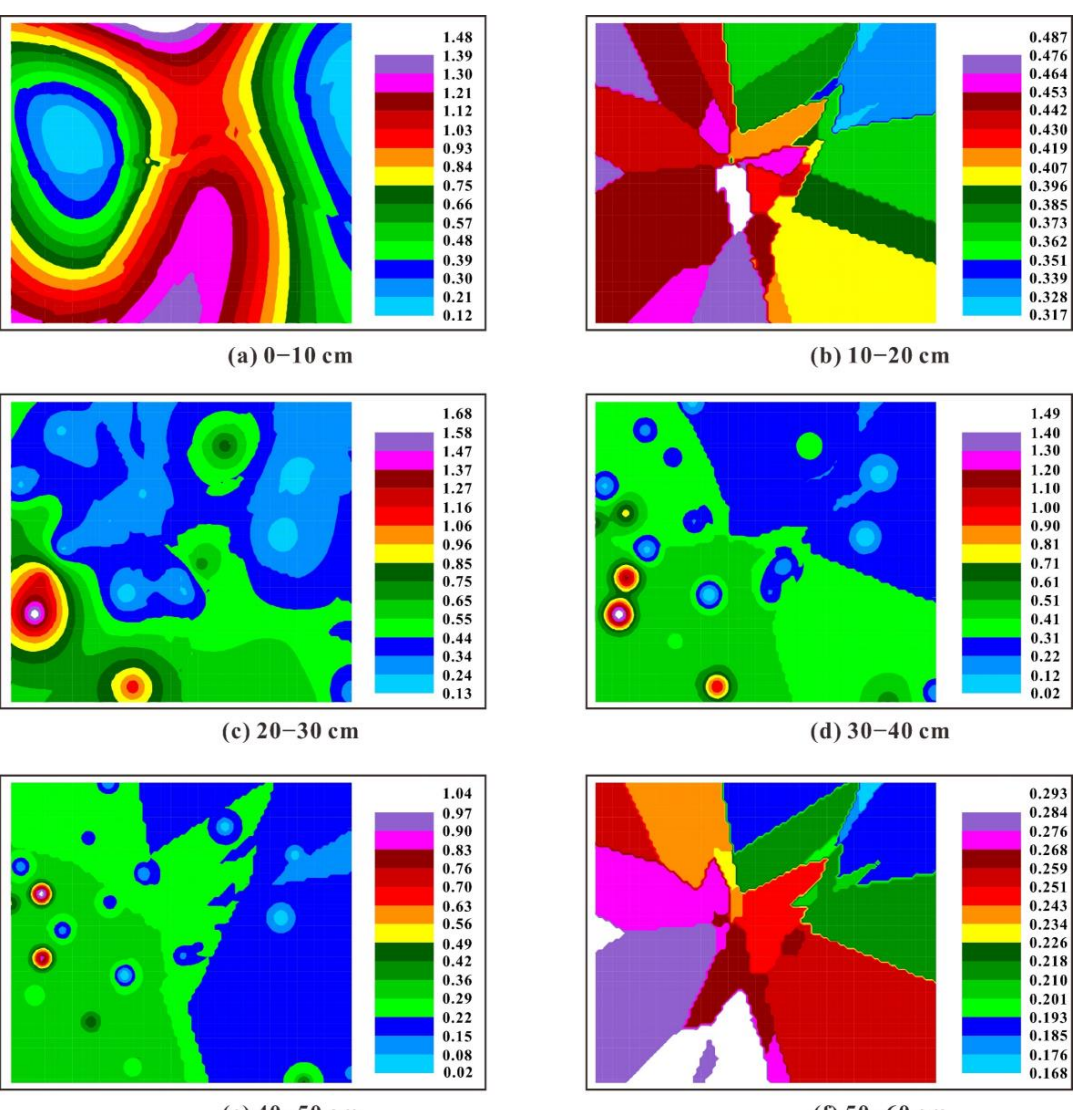
Spatial distribution maps of the Ks (m/day) in the South Dump based on the ordinary kriging interpolation. (**a**–**f**) represent the soil layers at different depths (0–10 cm, 10–20 cm, 20–30 cm, 30–40 cm, 40–50 cm and 50–60 cm).

The spatial interpolation maps of the K factor and Ks in different soil layers of the South Dump are shown in Figure 3 and Figure 4. The K factor in topsoil (0–10 cm) is more variable than that of other soil layers, which might result from the uneven input of external organic matter due to the variations in surface vegetation. For instance, the areas dominated by woody plants (e.g., *Robinia pseudoacacia*, *Pinus tabuliformis* and *Caragana korshinsk*) are characterized by lower K factor values compared with the areas dominated by herbaceous plants (e.g., *Incarvillea sinensis*, *Elymus dahuricus* and *Saussurea japonica*), indicating that the plant configuration modes can exert an essential influence on soil erodibility. The K factor generally presents a decreasing tendency from west to east at 0–10 cm and 50–60 cm, while the K factor seems not to show any spatial distribution pattern along latitude or longitude for other soil layers. Meanwhile, many high-K factor or low-K factor “points” are sporadically distributed across the study area, which results from the strong heterogeneity of the reconstructed soils triggered by the irregular mixing of various substances (e.g., rock fragments, soil particles and plant residues) [72]. Vertically, the K factor in topsoil (0–10 cm) is lower than that of other soil layers in most areas of the South Dump, which might be attributed to the relatively higher SOM content in topsoil. Previous studies have confirmed that the existence of SOM benefits the formation of anti-erosion soil aggregates and the increase in soil infiltration, which can reduce soil erosion and surface runoff [2,73,74]. Saturated hydraulic conductivity is regarded as one of the most important indicators for the mobility of solute in soils, and understanding the spatial distribution characteristics of hydraulic properties is necessary for land reclamation and revegetation [75]. Overall, the Ks presents a striped distribution pattern at 0–10 cm while showing a patchy pattern in other soil layers. Moreover, the Ks in topsoil is obviously higher than that in deeper soil layers. Interestingly, the spatial distribution patterns of Ks are opposite to those of soil bulk density (SBD) reported by Huang et al. [37]. Meanwhile, the K factor also shows opposite spatial distribution characteristics relative to the reported SBD at 50–60 cm. Generally, high SBD can lower soil permeability and increase the ability of soils to resist erosion processes, which might account for the opposite spatial distributions between K factor, Ks and SBD [23]. Moreover, the effects of soil properties on K factor and Ks can be further supported by Spearman’s correlation analyses. The results show that clay contents are positively correlated with the K factor (r = 0.332, *p* < 0.01) and negatively correlated with Ks (r = −0.615, *p* < 0.01), indicating that fine soil particles can improve soil erodibility and reduce saturated hydraulic conductivity. The spatial distribution patterns of the K factor and Ks in the South Dump highlight the significance of plant configuration modes and reconstructed soil properties (e.g., SBD, clay and SOM contents) on regulating soil erodibility, which can provide insight for vegetation configuration and soil management in similar ecosystems.

### 3.3. Effects of Soil Erodibility and Saturated Hydraulic Conductivity on Soil Nutrients

**Figure 5:**
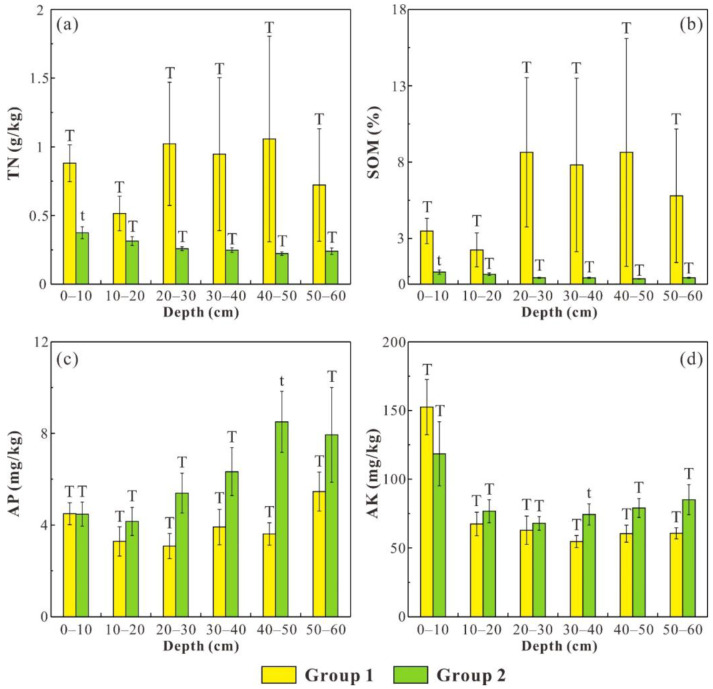
The average contents of TN (**a**), SOM (**b**), AP (**c**) and AK (**d**) in group 1 and group 2 (the classification of the two groups is based on the K factor values). The error bar represents the standard error and different forms of T denote significant difference.

**Figure 6:**
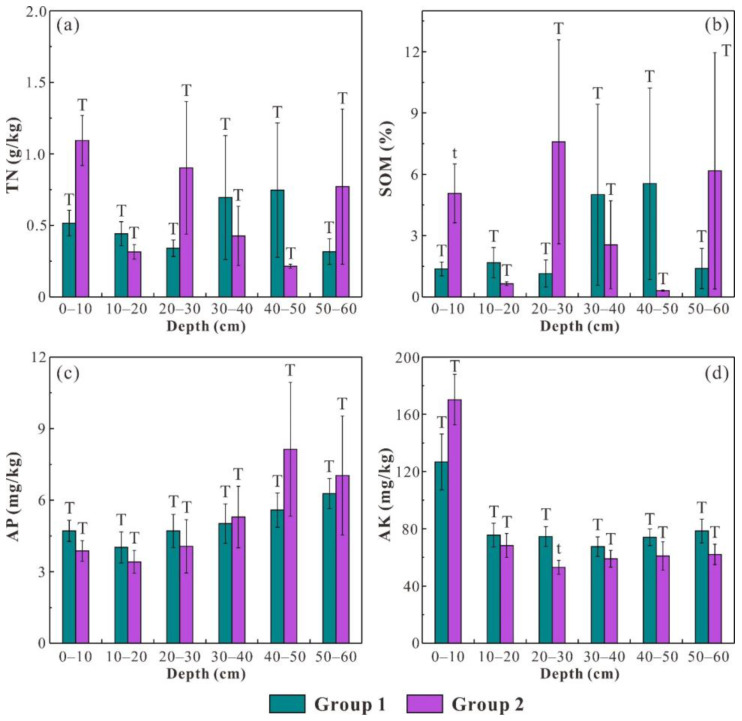
The average contents of TN (**a**), SOM (**b**), AP (**c**) and AK (**d**) in group 1 and group 2 (the classification of the two groups is based on the Ks values). The error bar represents the standard error and different forms of T denote significant difference.

To explore the effects of soil erodibility and saturated hydraulic conductivity on the distribution of soil nutrients (i.e., TN, SOM, AP and AK), soil samples were categorized into two groups (i.e., group 1 and group 2), and the independent-sample *t*-test was employed to compare the difference in soil nutrient contents between different groups. Group 1 comprises the samples whose K factor values (or Ks values) are lower than the average K factor (or average Ks), while group 2 is composed of the remaining soil samples (Figure 5 and Figure 6). The average TN and SOM contents in group 1 are obviously higher than those in group 2 in all soil layers, and the difference is significant (*p* < 0.01) at 0–10 cm (Figure 5a,b). Meanwhile, the average AP and AK contents in group 1 are obviously lower than those in group 2 in all soil layers (except for 0–10 cm) (Figure 5c,d). For the Ks classification scheme, the average AP content in group 1 is higher than that in group 2 at 0–30 cm, and the average AK content in group 1 is higher than that in group 2 at 10–60 cm (Figure 6c,d).

Generally, soil nutrients are always dissolved in soil solution or bound by soil particles, due to which they can be transported in dissolved and particulate form during soil erosion process [11,76]. Theoretically, dissolved fractions of soil nutrients can move both horizontally and vertically, and the vertical mobility of dissolved soil nutrients is mainly regulated by the leaching process that is largely determined by saturated hydraulic conductivity. Although available soil nutrients only account for a small proportion of the total nutrient content, their mobility and accumulation in soil profiles are of great significance because they are mostly dissolved in soil solution and can be easily utilized by plant roots [11]. By comprehensively analyzing the distribution patterns of soil nutrients among the two groups, it is reasonable to conclude that lower soil erodibility benefits the accumulation of TN and SOM in reconstructed soils, while AP and AK contents are largely regulated by saturated hydraulic conductivity. This conclusion can be further supported by Spearman’s correlation analysis, which denotes that the K factor is negatively correlated with TN (r = −0.362, *p* < 0.01) and SOM (r = −0.380, *p* < 0.01) while Ks is negatively associated with AP (r = −0.229, *p* < 0.01) and AK (r = −0.180, *p* < 0.05). The statistical results reflect that the mobility of TN and SOM is more controlled by surface lateral runoff during soil erosion processes, while the migration of AP and AK is mainly determined by the vertical leaching process in soil profiles.

### 3.4. Implications for Soil Management and Land Reclamation in Opencast Coalmine Areas

The kriging interpolation maps of the K factor and Ks can provide helpful guidance for the management practices of reconstructed soils and the spatial planning of land use during land reclamation in similar ecosystems. In the South Dump, the vegetation configuration modes should be optimized to improve the ability of reconstructed soils to resist erosion processes. Anti-erosion vegetation (e.g., black locust, shrub sophora and korshinsk peashrub) should be preferentially planted, and more exogenous organic materials (e.g., biochar and local organic fertilizer) should be exerted to reduce soil erodibility in high-K factor regions of the study area [23,31]. Moreover, it is quite important to sustain the soil fertility of reconstructed soils because the ability to maintain fertility is an essential indicator in evaluating the quality of land reclamation. The results of this study indicate that TN and SOM contents are mainly affected by the K factor, while AP and AK contents are more regulated by Ks. The influence of soil erodibility on TN and SOM can be mainly attributed to the soil erosion process, while there are many pathways through which saturated hydraulic conductivity affects the mobility of AP and AK. High Ks causes rapid water drainage and infiltration, which amplifies the leaching process of elements in soil profiles. Meanwhile, it also determines the abundance of air-filled porosity, which further exerts an important influence on the uptake of soil nutrients by plants [77]. This study demonstrates that different reclamation measures should be adopted to obtain good soil conditions with appropriate soil erodibility and saturated hydraulic conductivity for the conservation of different soil nutrients. This study can also benefit the improvement of the Chinese “completion standards on land reclamation quality” that have not yet considered soil erodibility or saturated hydraulic conductivity as indicators for land reclamation quality.

## 4. Conclusions

The effects of soil erodibility and saturated hydraulic conductivity on TN, SOM, AP and AK were determined, and spatial distribution maps of the K factor and Ks in different soil layers were presented based on geostatistical analyses. The main conclusions can be summarized as follows:

(1) The K factor in topsoil is obviously lower than that in other soil layers due to the external input of organic matter, while Ks generally tends to decrease along with soil depth. The K factor in middle soil layers has a strong spatial dependence and is mainly controlled by intrinsic factors, while Ks levels at 10–20 cm and 50–60 cm are mostly regulated by extrinsic factors based on the C_0_/(C_0_ + C) values.
(2) The statistical results indicate that K factor is negatively correlated with SOM (r = −0.380, *p* < 0.01) and TN (r = −0.362, *p* < 0.01), while Ks is negatively associated with AP (r = −0.229, *p* < 0.01) and AK (r = −0.180, *p* < 0.05). The phenomenon should be attributed to the fact that TN and SOM mainly exist in particulate form, while AP and AK are always dissolved in soil solutions, and their mobility is largely influenced by the leaching process in soil profiles. The intriguing results indicate that the conservation of different soil nutrients requires different reclamation measures.
(3) Based on the spatial information for the K factor and Ks, anti-erosion vegetation should be preferentially planted in high-K factor regions to reduce the soil erodibility in the South Dump. The results of this study can guide the spatial planning of land use and the implementation of land reclamation measures in the reclaimed land of similar ecosystems.

## Figures and Tables

**Table 1:** Statistical characteristics of the K factor and Ks in different soil layers.

Soil Depth (cm)	Mean	Median	Maximum	Minimum	CV (%)	K-S
K (t ha h (ha MJ mm)^−1^)						
0–10	0.0384	0.0368	0.0543	0.0203	24.20	0.95
10–20	0.0423	0.0441	0.0540	0.0279	17.65	0.81
20–30	0.0421	0.0452	0.0571	0.0228	24.21	0.66
30–40	0.0412	0.0409	0.0554	0.0255	21.68	0.98
40–50	0.0445	0.0453	0.0566	0.0270	16.42	0.60
50–60	0.0423	0.0417	0.0531	0.0213	18.46	0.96
Ks (m/day)						
0–10	0.689	0.392	2.278	0.094	94.24	0.07
10–20	0.430	0.331	1.184	0.046	76.90	0.62
20–30	0.496	0.204	1.955	0.032	105.13	0.12
30–40	0.387	0.227	1.506	0.014	104.18	0.27
40–50	0.256	0.169	1.051	0.010	106.35	0.12
50–60	0.245	0.154	0.876	0.006	94.75	0.34

**Table 2:** Parameters for the semivariogram analyses of the K factor and Ks in different soil layers.

Soil Depth (cm)	Optimal Model	C_0_/(C + C_0_) (%) ^a^	R^2 b^	RSS ^c^	A_0_/m ^d^
K (t ha h (ha MJ mm)^−1^)					
0–10	Gaussian	36.78	0.80	9.07 × 10^−10^	1143.15
10–20	Gaussian	0.17	0.92	3.47 × 10^−10^	211.31
20–30	Gaussian	0.08	0.85	7.63 × 10^−10^	145.49
30–40	Gaussian	0.12	0.64	1.95 × 10^−9^	148.96
40–50	Gaussian	0.17	0.65	5.16 × 10^−10^	143.76
50–60	Linear	100	0.66	1.96 × 10^−10^	649.43
Ks (m/day)					
0–10	Gaussian	13.82	0.85	1.40 × 10^−2^	2793.80
10–20	Linear	100	0.80	3.60 × 10^−3^	651.73
20–30	Exponential	17.12	0.94	2.41 × 10^−3^	795.00
30–40	Gaussian	0.06	0.83	4.14 × 10^−3^	226.90
40–50	Gaussian	0.13	0.61	7.02 × 10^−4^	164.54
50–60	Linear	100	0.81	1.24 × 10^−3^	631.17

^a^ Ratio of spatial heterogeneity, where C_0_ is Nugget and C + C_0_ is Sill; ^b^ Determination coefficient; ^c^ Residual sum of squares; ^d^ Range.

**Table 3:** Stability evaluation for different models of the ordinary kriging interpolation.

Soil Depth (cm)	Optimal Model	ME	AME	RMSE
K (t ha h (ha MJ mm)^−1^)				
0–10	Gaussian	4.86 × 10^−4^	7.15 × 10^−3^	8.94 × 10^−3^
10–20	Gaussian	−2.90 × 10^−4^	6.37 × 10^−3^	7.44 × 10^−3^
20–30	Gaussian	−1.67 × 10^−4^	9.45 × 10^−3^	1.11 × 10^−2^
30–40	Gaussian	−1.36 × 10^−4^	7.96 × 10^−3^	9.30 × 10^−3^
40–50	Gaussian	−1.18 × 10^−4^	5.86 × 10^−3^	7.57 × 10^−3^
50–60	Linear	6.70 × 10^−4^	5.86 × 10^−3^	6.75 × 10^−3^
Ks (m/day)				
0–10	Gaussian	−3.45 × 10^−2^	5.51 × 10^−1^	7.43 × 10^−1^
10–20	Linear	3.03 × 10^−3^	2.82 × 10^−1^	3.35 × 10^−1^
20–30	Exponential	−9.81 × 10^−3^	4.08 × 10^−1^	4.95 × 10^−1^
30–40	Gaussian	3.64 × 10^−5^	3.11 × 10^−1^	3.95 × 10^−1^
40–50	Gaussian	1.24 × 10^−2^	2.05 × 10^−1^	2.72 × 10^−1^
50–60	Linear	1.08 × 10^−2^	1.81 × 10^−1^	2.34 × 10^−1^

## Data Availability

Not applicable.
